# Research on the Characteristics of Heat Transfer and Mass Transfer of Imitation Heart Pulse Membrane Distillation

**DOI:** 10.3390/membranes16090295

**Published:** 2026-09-09

**Authors:** Xiaoxuan Zhu, Zhoujun Lan, Zhuang Song, Peng Wang, Derong Duan, Zhongyi Jiang

**Affiliations:** 1 School of Mechanical Engineering, University of Jinan, Jinan 250022, China; 2 School of Chemical Engineering and Technology, Tianjin University, Tianjin 300072, China; 3 Shandong Guoshun Co., Ltd., Jinan 250030, China

**Keywords:** membrane distillation, heartbeat-imitating pulsation, membrane modification, brackish water treatment

## Abstract

Membrane distillation, a separation technology, has drawn wide attention in industries such as seawater desalination and high-salinity wastewater treatment. However, conventional steady-flow operation suffers from flux decline and membrane fouling, while standard square-wave and sine-wave pulsations provide limited enhancement and stability. To address these issues, this study introduces heartbeat-mimicking pulsatile flow. Its effects on membrane distillation performance were evaluated through comparative (steady vs. pulsatile) experiments, a three-level orthogonal experiment, and tests with various feed solutions and modified membranes. Results show that, under baseline conditions, the heartbeat-mimicking pulsatile flow yielded a 17.5% higher average flux than steady flow, while its conductivity increase was only 25%, far below the 93% for steady flow. In the orthogonal experiment, the heartbeat-mimicking waveform accounted for the largest proportion of total variance (50.1%) among the tested parameters. Furthermore, compared to traditional sine or square waves, this biomimetic pulsation features unique acceleration-rest characteristics, making it highly applicable to complex feed solutions and effectively mitigating membrane fouling. This study aims to identify a new pulsation mode that can overcome the limitations of steady-flow membrane distillation.

## 1. Introduction

**Figure 1:**
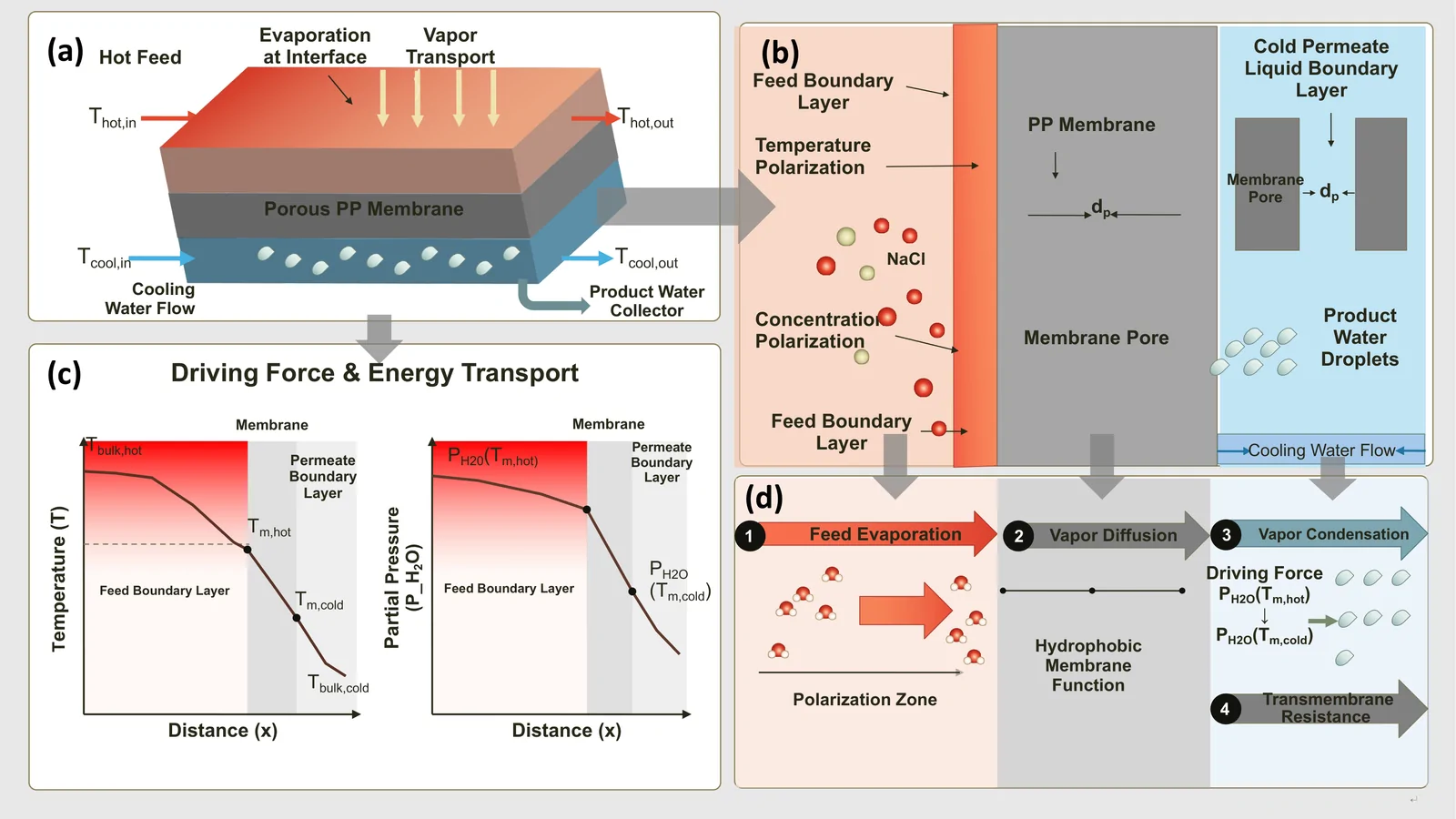
Schematic of the membrane distillation process: (**a**) macroscopic physical structure; (**b**) magnified microscopic interface of (**a**); (**c**) corresponding spatial driving force (temperature/pressure) gradients; (**d**) breakdown of mass transfer steps based on the interface in (**b**).

With continued global population growth and accelerating industrialization, freshwater scarcity has become a key constraint on sustainable development. Membrane distillation, a thermally driven membrane technology using low-grade heat, features low operating pressure, high rejection, and relatively low mechanical requirements for membrane materials, showing great promise for seawater desalination and high-salinity wastewater treatment [1,2,3], the schematic diagram of membrane distillation is shown in Figure 1.

Since its introduction in the 1960s, membrane distillation has evolved from fundamental mechanism studies to application development. Current research focuses on process intensification, membrane modification, and anti-fouling/anti-scaling.

In flow intensification, Liu et al. combined slip surfaces with pulsed flow to effectively mitigate scaling in direct contact membrane distillation (DCMD) [4]; Zhu et al. visualized geothermal brackish water membrane distillation from multiple perspectives, revealing how flow conditions regulate membrane fouling [5]. Pulsatile flow is an active enhancement method that periodically disrupts the boundary layer and reduces polarization effects, but conventional pulsation waveforms typically lack the acceleration–peak–deceleration–rest pattern characteristic of biological fluid flow. In recent years, biomimetic flow has become a research focus because it better aligns with natural fluid dynamics. Heartbeat-mimicking pulsatile flow, characterized by periodicity, unsteadiness, and low impact similar to cardiac pumping, can restore the boundary layer with small perturbations while preventing strong vibrations from damaging the membrane structure. However, while existing literature has successfully explored conventional pulsatile flows [4], systematic studies focusing specifically on the hydrodynamic application of biomimetic unsteady waveforms—which feature unique acceleration-rest characteristics, such as heartbeat-mimicking flows—in membrane distillation remain lacking.

As for membrane materials, hydrophobic membranes such as PP, PTFE, and PVDF are the core components of membrane distillation. Li et al., in a review of PVDF membrane distillation for water treatment, noted that PVDF membranes, owing to their good hydrophobicity and chemical stability, are among the most widely used in the field [6]. Li et al. fabricated a PVA-PAA/PTFE Janus membrane and tested it via DCMD with humic acid and CaSO_4_. The membrane achieved a flux of 16.2 kg/(m^2^·h) and showed significantly better resistance to organic fouling and scaling than the unmodified PTFE membrane [7]. Regarding membrane surface functionalization, Zhou et al. reviewed tannic acid (TA)-based modification, highlighting that TA, rich in phenolic hydroxyl groups, adheres firmly to membrane surfaces via hydrogen bonding, hydrophobic interactions, and metal coordination. As a natural plant extract, TA is far cheaper than dopamine and an ideal choice for green modification [8]. Zhang et al. fabricated an SBS-based superhydrophobic beaded membrane, achieving a flux of 47 kg/(m^2^·h) and stable performance over 192 h of seawater desalination [9].

These studies indicate that both hydrophilic modification and superhydrophobicity offer distinct advantages. However, most membrane modification research has been conducted under steady-flow conditions, and systematic performance comparisons under pulsatile flow are lacking. While fluid hydrodynamics and surface modifications are independently recognized as effective fouling control strategies [10], there remains a critical gap in systematically combining dynamic fluid shear with static surface chemistry [4]. Integrating heartbeat-mimicking pulsation with specific surface modifications promises synergistic optimization of fouling resistance and mass transfer flux.

Specifically, TA and SBS/SiO_2_ were selected to represent two distinct anti-fouling strategies: the hydrophilic TA isolates foulants via a hydration layer (ideal for calcium scaling), while the superhydrophobic SBS/SiO_2_ minimizes solid–liquid contact to delay wetting (ideal for hypersaline feeds).

The main research contents include:

(1) Verification of the dominant role of heartbeat-mimicking pulsation in heat and mass transfer during membrane distillation: waveform design and baseline performance measurement. Basic MD performance was measured under steady-flow conditions to establish a reference for comparison. A three-factor, three-level orthogonal experiment was then conducted with pulsation waveform, operating temperature, and NaCl concentration as factors, and the optimal parameter combination was determined via range analysis and ANOVA.
(2) Demonstration of the scenario-dependent performance of membrane materials under different conditions: preparation and performance evaluation of various modified membranes. Hydrophilic Janus membranes were prepared by direct TA immersion, and superhydrophobic membranes by SBS/SiO_2_ pulse ultrasonication-scraping. Under optimal pulsatile conditions, the mass and heat transfer performance of unmodified PP, TA hydrophilic-modified, and SBS/SiO_2_ superhydrophobic membranes was compared in wetting-prone (NaCl solution) and scaling-prone (simulated brackish water) environments.
(3) Analysis of the synergistic effect between pulsation and membrane modification. From a macroscopic perspective, the individual and synergistic effects of pulsation enhancement and membrane modification were quantified through comparative experiments under steady and pulsatile flow conditions using unmodified and TA-modified membranes. From a microscopic perspective, characterization of the three membranes formed a complete evidence chain with the macroscopic mass transfer results.

## 2. Materials and Methods

### 2.1. Materials and Reagents

The base membrane was a commercial polypropylene (PP) flat-sheet microporous membrane (Zhejiang Feite Filter Equipment Co., Ltd., Jiaxing, China) with an effective area of 0.0154 m^2^. The commercial PP membrane has a nominal pore size of 0.22 μm, a porosity of 85%, a thickness of 200 μm, an initial water contact angle of 130°, and a liquid entry pressure (LEP) generally >0.2 MPa. Before use, it was soaked in distilled water for 10 min and rinsed with deionized water (Beijing Watsons Distilled Water Co., Ltd., Beijing, China).

Major reagents are listed in Table 1.

### 2.2. Heartbeat-Mimicking Pulsatile Membrane Distillation Experimental Platform

**Figure 2:**
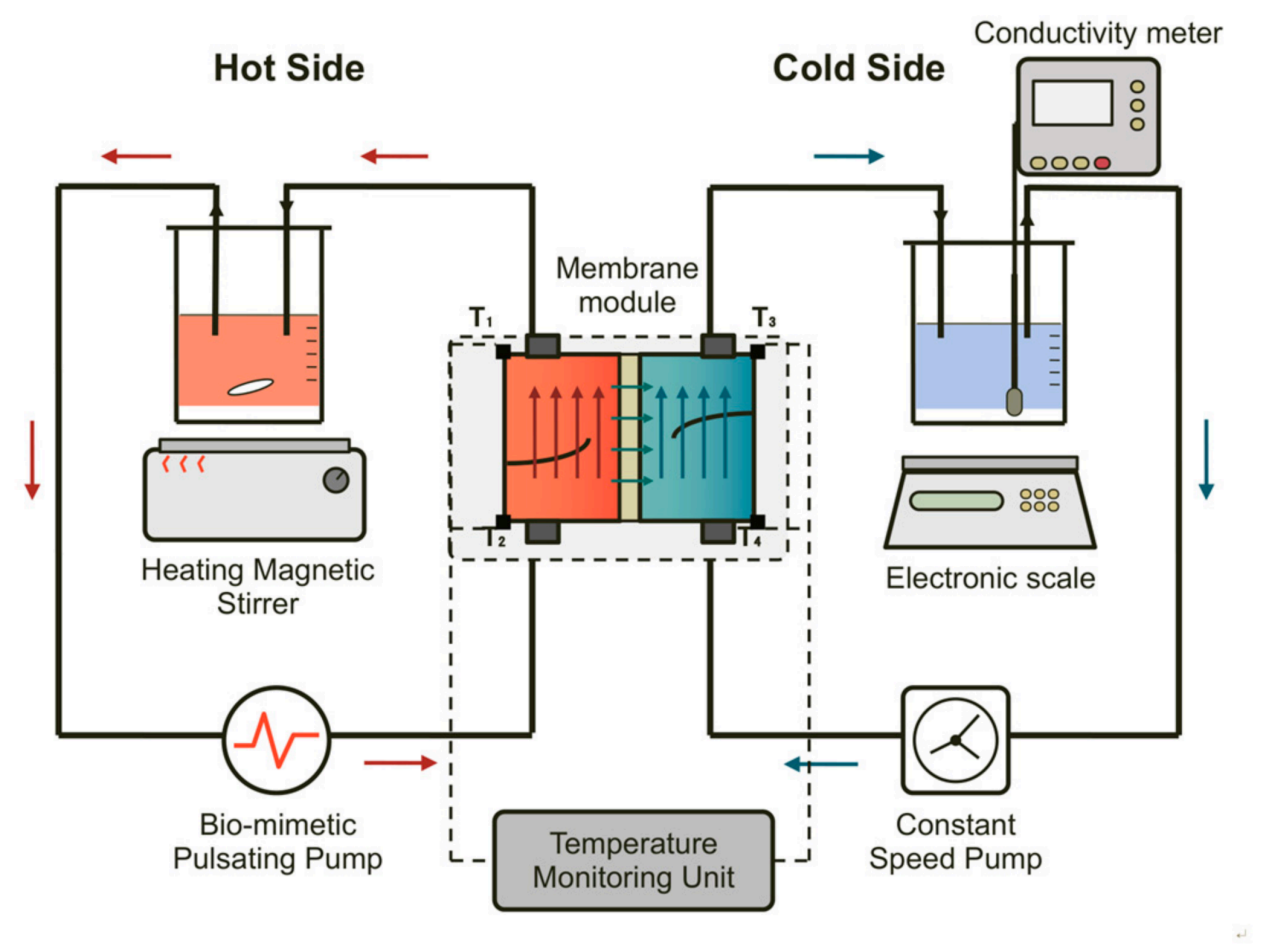
Schematic of the heartbeat-mimicking pulsatile membrane distillation experimental platform.

The experimental setup consists of a hot-side circulation unit, a cold-side circulation unit, a membrane module, a pulsation driving unit, and a data acquisition unit, the connection diagram of the apparatus is shown in Figure 2.

The hot-side circulation system is equipped with a constant-temperature water bath that maintains the feed temperature within ±0.5 °C. The cold side uses a circulating constant-temperature cooling system to keep the cold-side temperature stable, ensuring a stable transmembrane temperature. To generate a pulsatile flow mimicking the heartbeat, a programmable peristaltic pump was employed in the system. A Lab-X01F peristaltic pump equipped with a YZ15 pump head and soft tubing (1.6 mm wall thickness) was used (Baoding Geyue Electronic Technology Co., Ltd., Baoding, China). It enabled cyclic switching of multiple flow-rate segments and time parameters, thereby generating a heartbeat-like pulsatile flow. Pulsation frequency is determined by the total cycle duration, amplitude by the difference between maximum and minimum rotational speeds, and the diastolic and arrest periods by the lengths of different time segments.

The data acquisition system included: an electronic balance (0.5 g resolution) for recording permeate mass; a 6-channel thermocouple recorder for real-time monitoring of inlet/outlet temperatures on both hot and cold sides; and a portable conductivity meter for periodic permeate conductivity measurement [11].

### 2.3. Feed Solution Systems

In the feed solution system, NaCl solutions at 10, 35, and 60 g/L were prepared to represent typical brackish water(e.g., representative values in the Ludong region) [5], average seawater [2], and concentrated hypersaline brine [9], respectively, for orthogonal experiments.

According to the literature [1], simulated brackish water with TDS ≈ 7.7 g/L was prepared following the formulation in Table 2. After preparation, the solution was left to settle, and the supernatant was used.

### 2.4. Membrane Modification Method

#### 2.4.1. TA Hydrophilic Modified Membrane (Simplified Immersion Method)

The anhydrous ethanol pretreatment significantly improved the surface wettability of the chemically inert PP membrane. TA, being rich in phenolic hydroxyl groups, adheres firmly to the PP matrix primarily via hydrophobic interactions (aromatic rings) and intermolecular hydrogen bonding [8]. Unlike multi-component TA/metal-ion cross-linking systems that rely on strong coordination bonds and require very low concentrations (e.g., 1–2 g/L), this single-component immersion method relies entirely on physical adsorption. Therefore, a relatively high TA concentration (0.50 g/50 mL, approx. 10 g/L) was selected based on our preliminary optimization. This concentration ensures sufficient molecular supply to achieve saturated, continuous hydrophilic coverage without causing physical accumulation that blocks membrane pores. Furthermore, the immersion time of 2 h was determined via preliminary wetting tests to be the minimum time required to reach a stable adsorption equilibrium, ensuring that water droplets can completely spread on the membrane surface within 5 s.

0.50 g TA was dissolved in 50 mL deionized water with manual stirring for 5 min until fully dissolved (light brown, transparent). Four PP membranes (13 cm × 13 cm) pretreated with anhydrous ethanol were laid flat in a tray (plastic-lined to avoid metal contact). TA solution was evenly poured over both sides of each membrane, and gloves were used to press the membranes to ensure complete wetting. The membranes were aligned, stacked, wrapped in cling film, and left at room temperature for at least 2 h. After removal, the membranes were gently rinsed with deionized water 2–3 times and stored for later use. Quality criterion: water droplets completely spread on the membrane surface within 5 s.

#### 2.4.2. SBS/SiO_2_ Superhydrophobic Modified Membrane (Pulse Ultrasonication-Scraping Method)

0.33 g nano-SiO_2_ was stirred with 7 mL anhydrous ethanol into a paste, then added to 50 mL ethyl acetate and pre-mixed by magnetic stirring for 5 min. Using an ultrasonic disruptor (Qiaoniu Technology, 35% power) in pulse mode (on 2 s/off 6 s) with a total cumulative ultrasonic time of 15 min, a uniform milky white suspension was obtained. 1.0 g SBS was added in portions and dissolved by magnetic stirring for 1–2 h, yielding a translucent viscous solution. The PP membrane was laid flat in a container and uniformly scraped in one pass with a glass rod to a wet film thickness of 50–100 μm. The coated membrane was left to dry in a ventilated indoor area for 30 min, then gently rinsed with deionized water to remove weakly adhered particles and air-dried. Quality criterion: static water contact angle ≥140°, with water droplets rolling off at a tilt angle of 5–10°.

These specific modification parameters were carefully optimized to balance coating integrity and vapor mass transfer. The SBS concentration (20 g/L) and nano-SiO_2_ dosage (6.6 g/L, maintaining a 3:1 mass ratio) were selected based on related superhydrophobic membrane literature [9] to balance solution viscosity and micro/nano-roughness construction. During preparation, the pulse ultrasonication mode (2 s on/6 s off) at 35% power was specifically adopted to control the temperature of the ethyl acetate solvent (boiling point 77 °C) strictly below 40 °C. This prevented rapid solvent volatilization while ensuring the complete elimination of visible SiO_2_ agglomerates within the 15-min cumulative duration. Finally, a wet scraping thickness of 50–100 μm was selected, which naturally shrinks to a 10–30 μm dry layer after solvent evaporation. This thickness guarantees complete coverage of the PP substrate to prevent wetting while avoiding the formation of an overly dense barrier that would drastically reduce MD flux.

**Figure 3:**
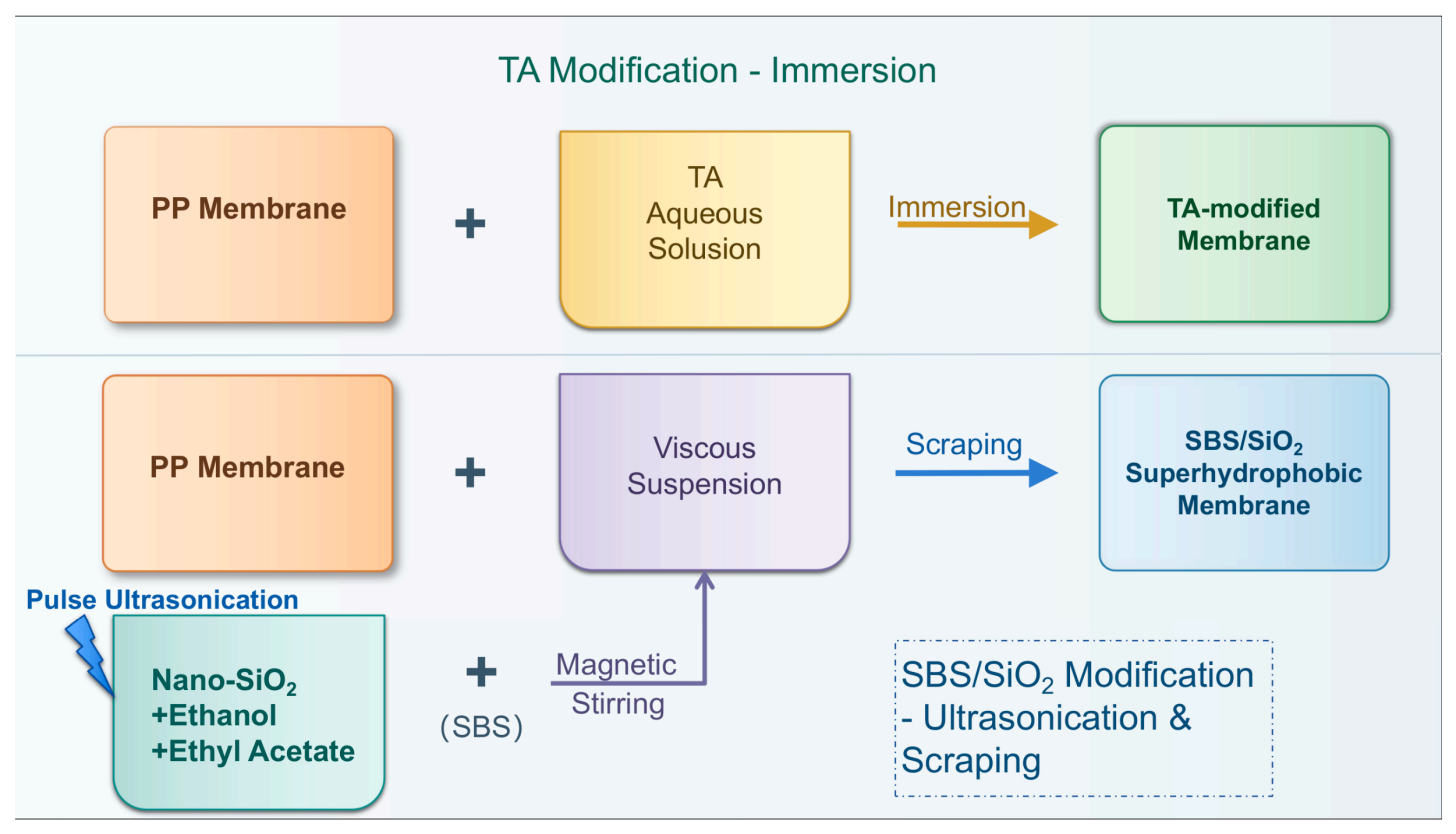
Schematic about the membrane modification methods.

A schematic illustration about the membrane modification methods is shown in Figure 3.

### 2.5. Experimental Design

#### 2.5.1. Orthogonal Experimental Design

Pulsation waveform (A), operating temperature (B), and NaCl concentration (C) were selected as factors, each at three levels. Experiments were arranged using an L_9_ orthogonal array [12], as shown in Table 3.

Average membrane flux (total permeate mass in 60 min/membrane area/time) and flux decline rate were used as evaluation indicators. Range analysis and ANOVA were employed to determine the significance order of factors and the optimal level combination.

#### 2.5.2. Waveform Design

This study designed three heartbeat-mimicking pulsatile waveforms, with their time parameters listed in Table 4.

The specific segment settings for flow rate variation in each waveform were programmed into the peristaltic pump controller during experiments. The resting waveform mimics a normal heart rate, with a balanced systole-to-diastole ratio; The exercise waveform simulates tachycardia with doubled systolic frequency and shortened pause; the arrhythmia waveform simulates a compensatory pause, inserting a longer rest (0.3 s) after a complete pulsation to maximize thermal boundary recovery.

#### 2.5.3. Comparative Experiment on Modified Membrane Performance

Based on the optimal pulsatile conditions determined by the orthogonal experiment, the following comparative experiments were conducted (as detailed in Section 3):

Section 3.2.1: Comparison of flux and conductivity for modified membrane, TA hydrophilic modified membrane, and SBS/SiO_2_ superhydrophobic modified membrane in NaCl solution.

Section 3.2.2: Long-term performance comparison of three membranes in simulated brackish water.

Section 3.3: Quantitative analysis of the synergistic effect between unmodified and TA-modified membranes under steady and pulsatile flow conditions.

It is important to note that to accurately capture transient boundary layer dynamics and continuous scaling behaviors, all experiments in this study (including the L_9_ orthogonal array) were designed as continuous, real-time dynamic mass tracking runs rather than independent end-point replicates. Consequently, single-run point estimates were utilized, and formal standard deviations or error bars could not be calculated for these specific transient data points. Rigorous statistical replicate measurements will be incorporated in future scale-up studies to further evaluate measurement variability.

### 2.6. Performance Indicators

Performance evaluation indicators are shown in Table 5.

Thermal efficiency is evaluated by the gained output ratio (GOR) and specific energy consumption, where STEC ≈ 650/GOR (kWh/m^3^).

## 3. Results and Discussion

### 3.1. Confirmation of Basic Performance and Parameter Optimization

#### 3.1.1. Confirmation of Basic Performance

**Figure 4:**
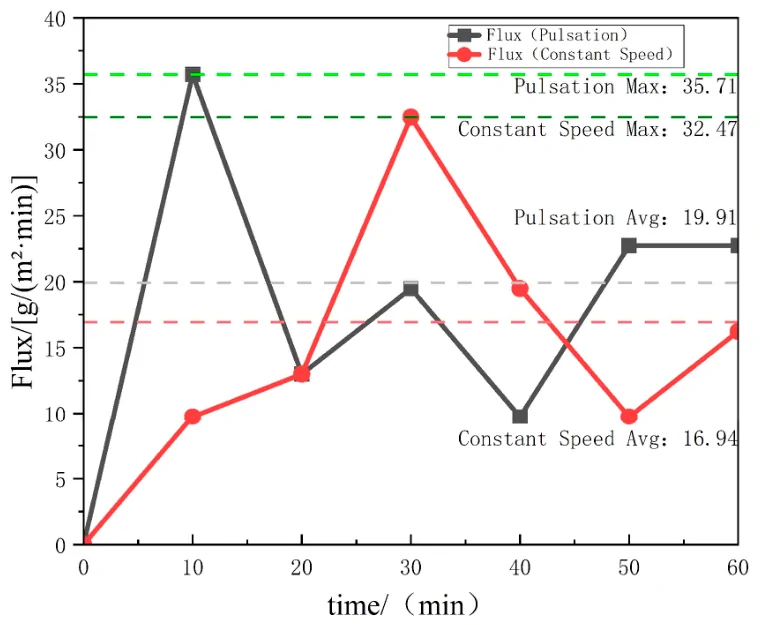
Membrane flux under constant-temperature conditions.

Using 35 g/L NaCl solution as feed, steady (150 rpm) and pulsatile (A2 exercise waveform) conditions were operated at 60 °C hot side and 20°C cold side for 60 min each. Results are shown in Table 6 and Figure 4.

From Table 6, the average flux under pulsatile conditions was about 17.5% higher than that under steady conditions, and the initial flux reached as high as 35.71 g/m^2^·min, far exceeding the 9.74 g/m^2^·min recorded under steady conditions. Pulsatile flow generated a strong scouring effect on the boundary layer early in the run, suppressing temperature polarization and markedly increasing the initial flux. The unusually high initial flux under pulsatile flow is mainly attributed to the intense initial fluid shear stress acting on the fresh, unfouled membrane surface, which rapidly strips away the developing thermal boundary layer before a stable polarization profile can be established. However, as shown in Figure 4, pulsation exhibited greater variation than the steady condition, so further parameter optimization is needed to achieve stable, high-efficiency pulsatile flow.

#### 3.1.2. Parameter Optimization

According to the orthogonal experimental scheme in Table 7, nine runs of 60 min each were conducted, and the average flux was recorded as the evaluation indicator. The orthogonal array and run numbers are shown in Table 7.

ANOVA was performed on the average flux of the nine runs, and the results are shown in Table 8. Since the L_9_(3^4^) orthogonal array had no empty column for error estimation, the factor with the smallest F value (B: temperature) was pooled into the error term, and its mean square was used as the error estimate.

From the ANOVA results in Table 8, the following conclusions can be drawn:

(1) The F values for pulsation waveform (A), temperature (B), and NaCl concentration (C) all fell below the critical value F_0.10_(2,2) = 9.00, indicating no statistical significance at the 10% level. This is mainly because the orthogonal experiment included only nine runs, leaving only two degrees of freedom for error and resulting in low test power. Furthermore, since these experiments were designed as continuous dynamic single-runs to track real-time mass transfer rather than independent end-point replicates, performing additional standard statistical evaluations such as t-tests was precluded. However, based on the sum of squares (SS), the contribution ranking is: pulsation waveform > temperature > NaCl concentration, which is fully consistent with the factor significance order from the subsequent range analysis.
(2) Since no factor reached statistical significance in the ANOVA, the optimal level was determined by visually comparing the mean flux at each factor level. A2 gave the highest mean flux of 33.91 g/(m^2^·min), B3 gave the highest mean flux for factor B at 32.83 g/(m^2^·min), and C3 the highest for factor C at 32.11 g/(m^2^·min)Therefore, the optimal combination is A2B3C3 (A2 waveform + 80 °C + 60 g/L NaCl), which was not included in the orthogonal array and requires supplementary verification.

#### 3.1.3. Flux Decline Characteristics of the Orthogonal Experimental Groups

**Figure 5:**
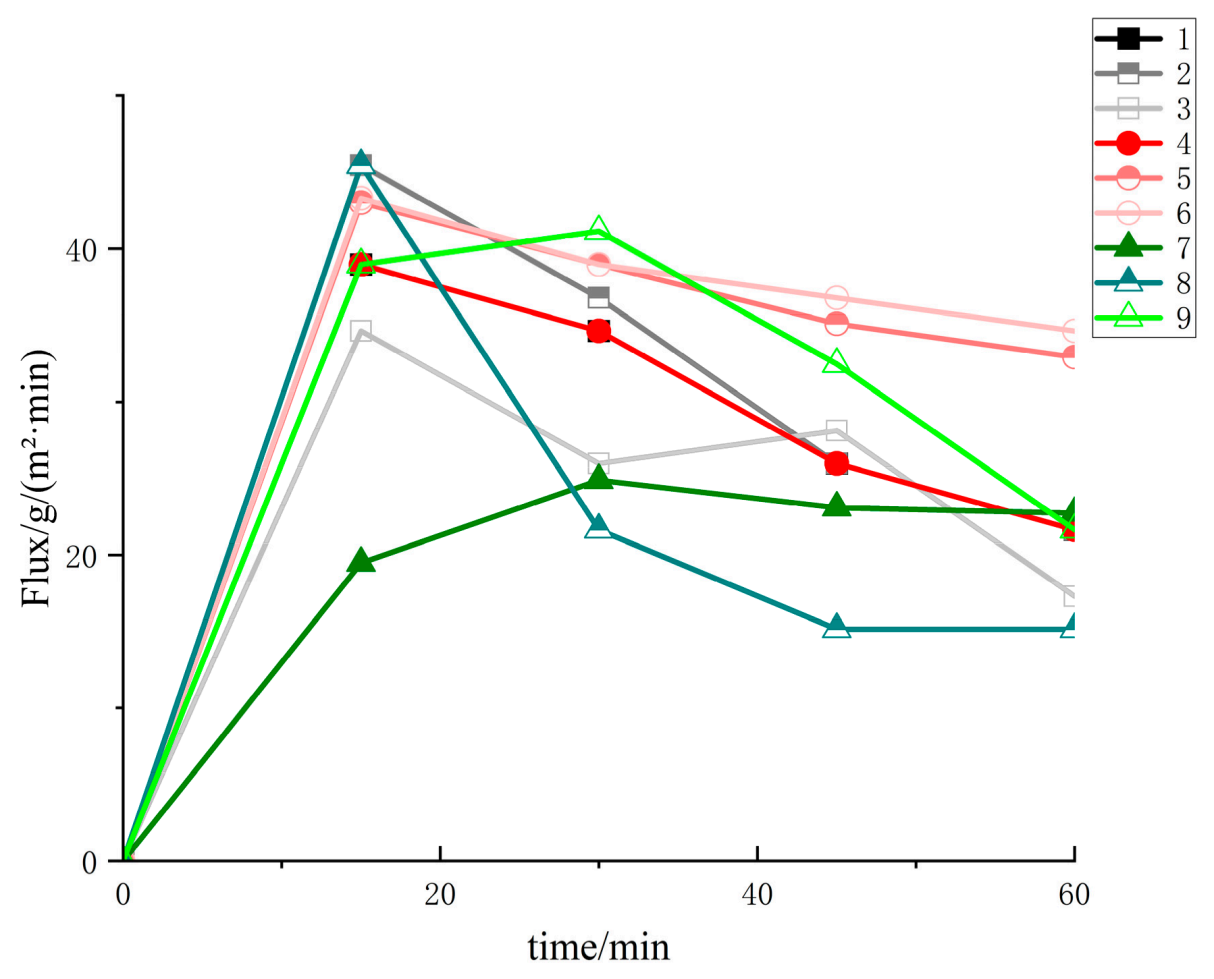
Flux–time curves of orthogonal experiments.

The flux decline over 60 min for each group under different waveform, temperature, and concentration combinations is shown in Figure 5. Overall, the flux of all groups decreased rapidly in the initial stage (0–15 min) and then leveled off from 15 to 60 min. Group 6 (A2B3C1) consistently maintained the highest flux and the slowest decline, indicating that the combination of high-frequency pulsation and low-concentration feed is favorable for sustaining high mass transfer efficiency.

To further quantify the decline, the 60-min flux decline rate was calculated for each group. Results are shown in Table 9.

In the table, initial flux is taken from the first 15-min interval, and final flux from the last 15-min interval. During these intervals, all other physical parameters governing the flux calculation—specifically the membrane’s effective mass transfer area (A = 0.0154 m^2^) and the time interval (Δt = 15 min)—were kept strictly constant. Decline rate = (initial flux − final flux)/initial flux × 100%.

Table 9 shows that:

(1) The two groups with the lowest flux decline rates were Experiment 5 (23.4%) and Experiment 6 (20.0%), both using the A2 exercise waveform. This finding corroborates the range analysis result that A2 gave the highest average flux, indicating that the high-frequency scouring of the exercise waveform continuously cuts the boundary layer, which not only elevates the initial flux but also slows flux decline to some extent.
(2) The highest flux decline rate (66.7%) occurred in Experiment 7 using the A3 arrhythmia waveform. Although the long pause favored thermal boundary layer recovery, concentration polarization accumulated during the quiescent phase, rendering subsequent flushing ineffective against the established concentration boundary layer and causing rapid flux decline.
(3) Comparing Experiment 3 (A1 B3 C2, 50.0% decline) and Experiment 5 (A2 B2 C2, 23.4% decline), both shared the same NaCl concentration (35 g/L) and similar operating temperatures (80°C vs. 70°C). Thus, the difference in flux decline rate is primarily attributed to the pulsation waveform. Compared with the A1 resting waveform, the A2 high-frequency short-cycle pulsation more effectively suppressed the growth of the concentration polarization layer.
(4) Within the A2 waveform, the flux decline rates of Experiment 5 (35 g/L) and Experiment 6 (10 g/L) were 23.4% and 20.0%, respectively. It can be seen that the lower the feed concentration, the smaller the flux decline, which is consistent with the fundamental rule that concentration polarization is weaker for low-concentration feeds in membrane distillation.
(5) It is worth noting that Experiment 7 (A3·B1·C2) exhibited a negative decline rate. This is because the combination of the lowest temperature (60 °C) and the long 0.3 s pause of the arrhythmia waveform delayed the establishment of a stable thermal driving force, causing an initially low flux that gradually increased as the system reached thermal equilibrium.
(6) It should be noted that the identical transient flux values (e.g., exactly 38.96, 34.63, or 21.65 g/(m^2^·min)) appearing in Table 9 are a mathematical quantization effect rather than reused datasets. As stated in Section 2.6, the mass increment is recorded as discrete steps (e.g., exactly 9.0 g, 8.0 g, or 5.0 g), naturally resulting in recurring calculated flux values for specific intervals. However, the total accumulated permeate mass over the entire 60-min cycle varies continuously, leading to distinct overall average flux values across different runs (as shown in Table 7, e.g., 30.30 vs. 33.55 g/(m^2^·min). This distinct average flux thoroughly confirms the statistical independence of each experimental dataset.

### 3.2. Performance Comparison of Membrane Distillation Under High-Salinity and Scaling-Prone Conditions

To explore the selectivity of heartbeat-mimicking pulsatile flow and membrane modification, this chapter systematically compared the mass transfer of unmodified PP, TA hydrophilic-modified, and SBS/SiO_2_ superhydrophobic membranes under wetting-prone (60 g/L NaCl) and scaling-prone (simulated brackish water) conditions, based on the optimal pulsatile conditions.

All experiments in this section shared the following optimal conditions: A2 exercise waveform (0.4 s period, 0.1 s pause), effective membrane area 0.0154 m^2^, data recorded every 15 min. NaCl solution tests were run at 80°C for 60 min; brackish water tests at 80°C for 90 min to cover both the induction and growth stages of scaling.

#### 3.2.1. Performance Comparison of Different Modified Membranes in NaCl Solution

In a 60 g/L NaCl feed solution, the main limiting factors are the decline in transmembrane vapor pressure difference and the risk of membrane pore wetting. This section compares flux levels, decline trends, and permeate conductivity of the three membranes, evaluating the applicability of hydrophilic and superhydrophobic modifications in high-salinity systems.

**Figure 6:**
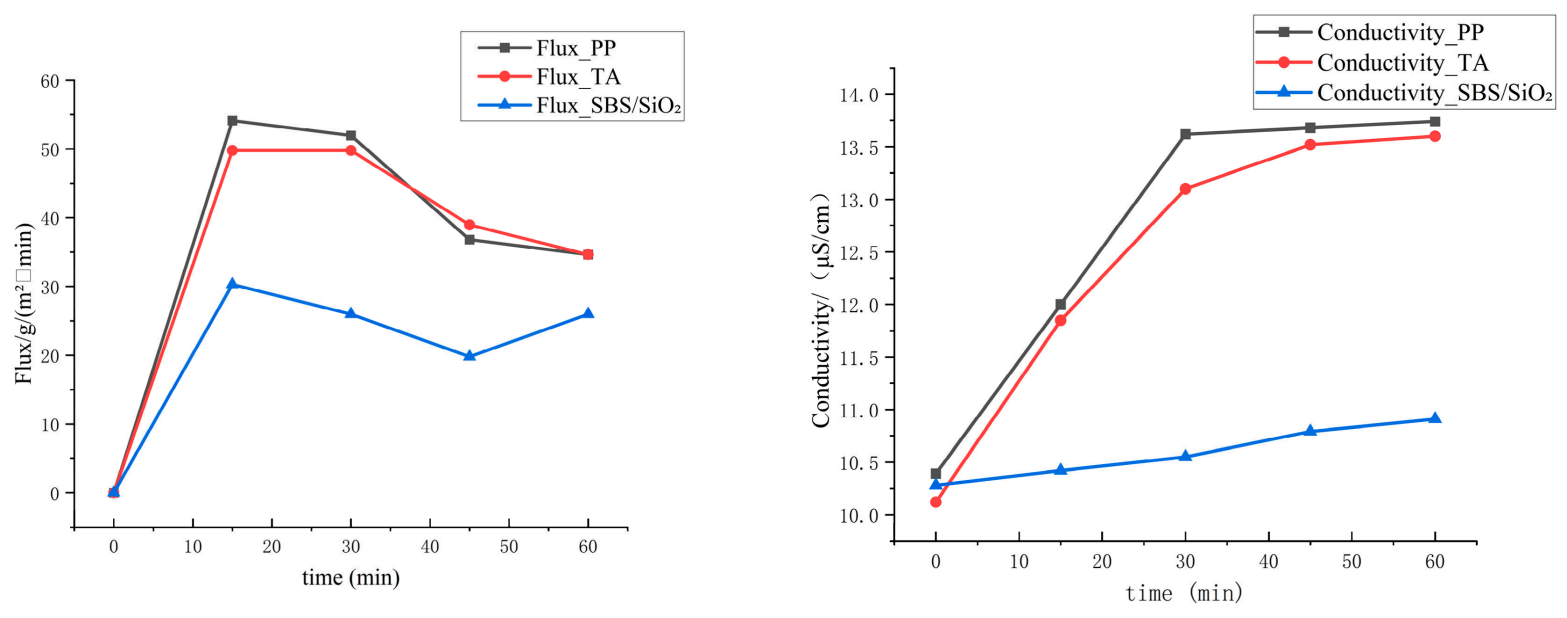
Flux and conductivity–time curves of the three membranes in NaCl solution.

Key performance indicators of the three experiments are summarized in Table 10, with the flux–time and conductivity–time curves shown in Figure 6.

Based on Table 10 and Figure 6, the following conclusions can be drawn:

The unmodified PP and TA membranes exhibited similar high flux levels (average fluxes of 44.37 and 43.29 g/(m^2^·min), respectively). This indicates that although the TA coating introduced an approximately 1 μm thick hydrophilic layer and added minor mass transfer resistance, the flux loss remained acceptable, while the surface hydration layer effectively retarded concentration polarization, reducing the flux decline rate from 36.0% to 30.4%.

In contrast, the SBS/SiO_2_ superhydrophobic membrane showed the lowest average flux—only 57.3% of the unmodified membrane—because its thick coating (50–100 μm) greatly increased vapor transport resistance across the membrane. However, in terms of permeate quality, the SBS/SiO_2_ membrane outperformed the others, with a final conductivity increase of only 6.1%, far below the 32.2% of the unmodified membrane. This confirms that its surface micro-/nano-rough structure successfully achieved excellent anti-wetting performance in high-salinity environments.

Under single-stage conditions without heat recovery, the gained output ratio (GOR) of the TA and unmodified PP membranes remained stable between 0.10 and 0.12, indicating that the TA surface modification did not adversely affect the system’s macroscopic thermodynamic balance or energy consumption. The superhydrophobic membrane had the lowest GOR (0.063), mainly because its thicker coating reduced the flux to about 60% of the other two membranes, with most of the input heat lost as sensible heat. The specific data are shown in Table 11.

#### 3.2.2. Performance Comparison of Different Modified Membranes in Brackish Water

Using simulated brackish water as feed, the anti-scaling performance of unmodified PP, TA hydrophilic-modified, and SBS/SiO_2_ superhydrophobic membranes was compared under optimal pulsatile conditions to verify the practical effectiveness of membrane modification in complex water matrices. The run time was extended to 90 min to fully cover both the scaling induction and growth stages, ensuring complete demonstration of the anti-scaling differences among the membranes.

**Figure 7:**
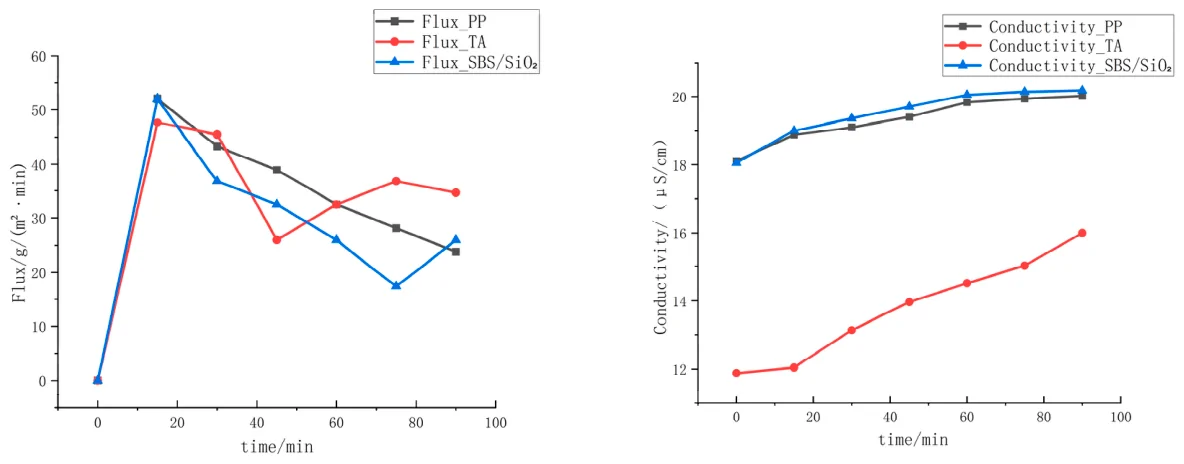
Flux and conductivity–time curves of the three membranes under brackish water conditions.

Key performance indicators of the three membranes are summarized in Table 12, and the flux–time and conductivity–time curves are shown in Figure 7.

From the experimental results:

When the feed was switched to simulated brackish water rich in scaling-prone ions such as Ca^2+^ and Mg^2+^, the performance of the three membranes reversed notably.

During the 90-min run, the TA hydrophilic modified membrane outperformed the unmodified PP membrane in average flux, with a flux decline rate strictly limited to 27.3%—only half that of the unmodified membrane (54.1%). The SBS/SiO_2_ membrane showed a flux similar to that of the unmodified membrane, better than its performance in high-salinity conditions. This finding clearly demonstrates the scenario-dependent nature of membrane modification strategies.

The GOR values of the TA and unmodified PP membranes were very close to those in the NaCl system, while the GOR of the SBS/SiO_2_ membrane was better than its performance in NaCl. All three membranes remained stable within 0.10–0.12, with none causing negative perturbation to the system’s macroscopic thermodynamic balance or energy consumption.The specific data are shown in Table 13.

### 3.3. Synergistic Effects and Microscopic Anti-Scaling Mechanism Analysis

Based on these findings, this section further elucidates the synergy between heartbeat-mimicking pulsatile fluid dynamics and the surface chemistry of modified membranes through cross-over experiments and SEM characterization.

#### 3.3.1. Synergy Analysis

**Figure 8:**
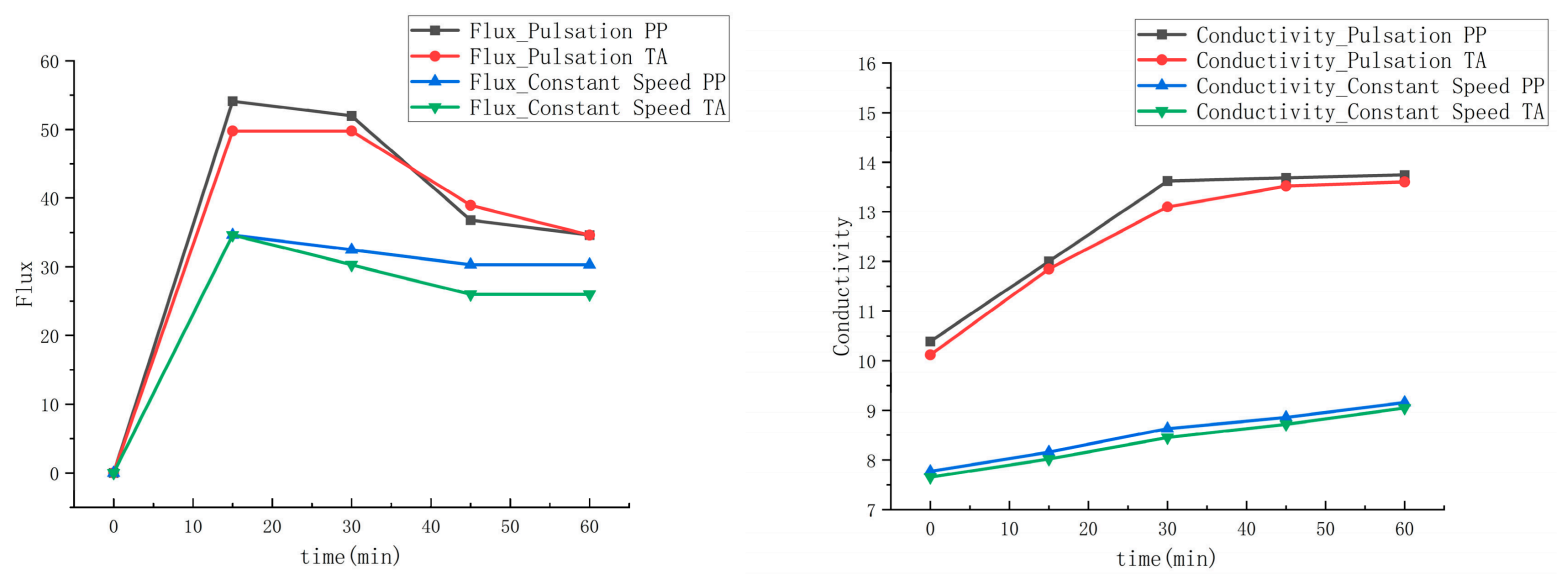
Flux and conductivity–time curves for the four experimental groups.

With TA hydrophilic modified membrane as the primary subject, MD performance under pulsatile and steady modes was compared, using the unmodified membrane as a control, to quantify the individual and synergistic effects of pulsation and membrane modification. Steady operation was at 150 rpm; other conditions were identical to those above (60 g/L NaCl, 80 °C). Key performance indicators of the four experiments are summarized in Table 14 and Figure 8.

The synergistic effect calculations are summarized in Table 15.

In the table, J_0_, J_1_, J_2_, and J_3_ denote the membrane flux under unmodified + steady, unmodified + pulsatile, TA-modified + steady, and TA-modified + pulsatile conditions, respectively. The plus/minus signs quantify the change in mass transfer performance upon adding each factor. Example: J_1_ − J_0_ = +12.40, indicating that pulsation alone exerted a positive effect on membrane flux, with a value of 12.40 g/m^2^·min.

The calculated synergistic effect was +1.67. It is tactfully acknowledged that this arithmetic calculation is relatively simple. Due to the lack of replicate experiments in this initial dynamic tracking study, a rigorous statistical variance analysis for this specific synergistic value was not feasible. Therefore, the +1.67 value is presented here as a preliminary deterministic observation, with strict replicate validation committed for future work. Nevertheless, this positive value indicates a favorable synergy between pulsatile flow and TA hydrophilic modification. Together they improved the flux by +11.32, exceeding the sum of their individual contributions (+9.65). Pulsation alone contributed +12.40, playing the dominant role in flux enhancement. In steady flow, the TA coating introduced some mass transfer resistance (−2.75), while periodic renewal of the hydration layer provided additional anti-fouling performance. This aligns with the finding in Section 3.2.2 that the TA membrane showed only a 27.3% flux decline in brackish water, far below the 54.1% of the unmodified membrane, confirming that the TA coating trades a certain amount of flux for anti-fouling capability under steady flow.

To quantitatively evaluate the energy consumption increment between the pulsatile and steady modes, the heat transfer efficiencies of the representative four cross-over groups were calculated (Table 16). The effective evaporation heat under pulsatile conditions (0.026 kW) is approximately 1.4–1.5 times that of the steady conditions (0.017–0.019 kW), clearly reflecting the enhancement of evaporation by pulsation. However, the temperature drop across the module in the pulsatile mode reaches 12–13 °C, leading to a sensible heat loss roughly twice that of the steady mode (which has a temperature drop of only 5–6 °C). Consequently, the overall GOR under pulsatile flow (0.11–0.12) is lower than that under steady flow (0.18–0.20). It is important to note that the seemingly higher GOR in the steady mode is somewhat of an artifact caused by its extremely low permeate flow rate (low water production). Furthermore, the difference in thermal efficiency between the TA-modified and unmodified membranes is minimal, indicating that the hydrodynamic operating mode (steady vs. pulsatile) impacts the macroscopic energy consumption far more significantly than the membrane surface chemistry.

#### 3.3.2. SEM Mechanism Analysis

To explain the differences in flux decline, anti-fouling, and anti-wetting among the membranes in NaCl solution and simulated brackish water, the commercial original PP membrane, used PP membrane, TA-modified membrane, and SBS/SiO_2_ superhydrophobic membrane were characterized by scanning electron microscopy (SEM). The morphological results were highly consistent with the previously reported macroscopic MD performance.

(1) PP membrane: continuous scaling and physical blocking

**Figure 9:**
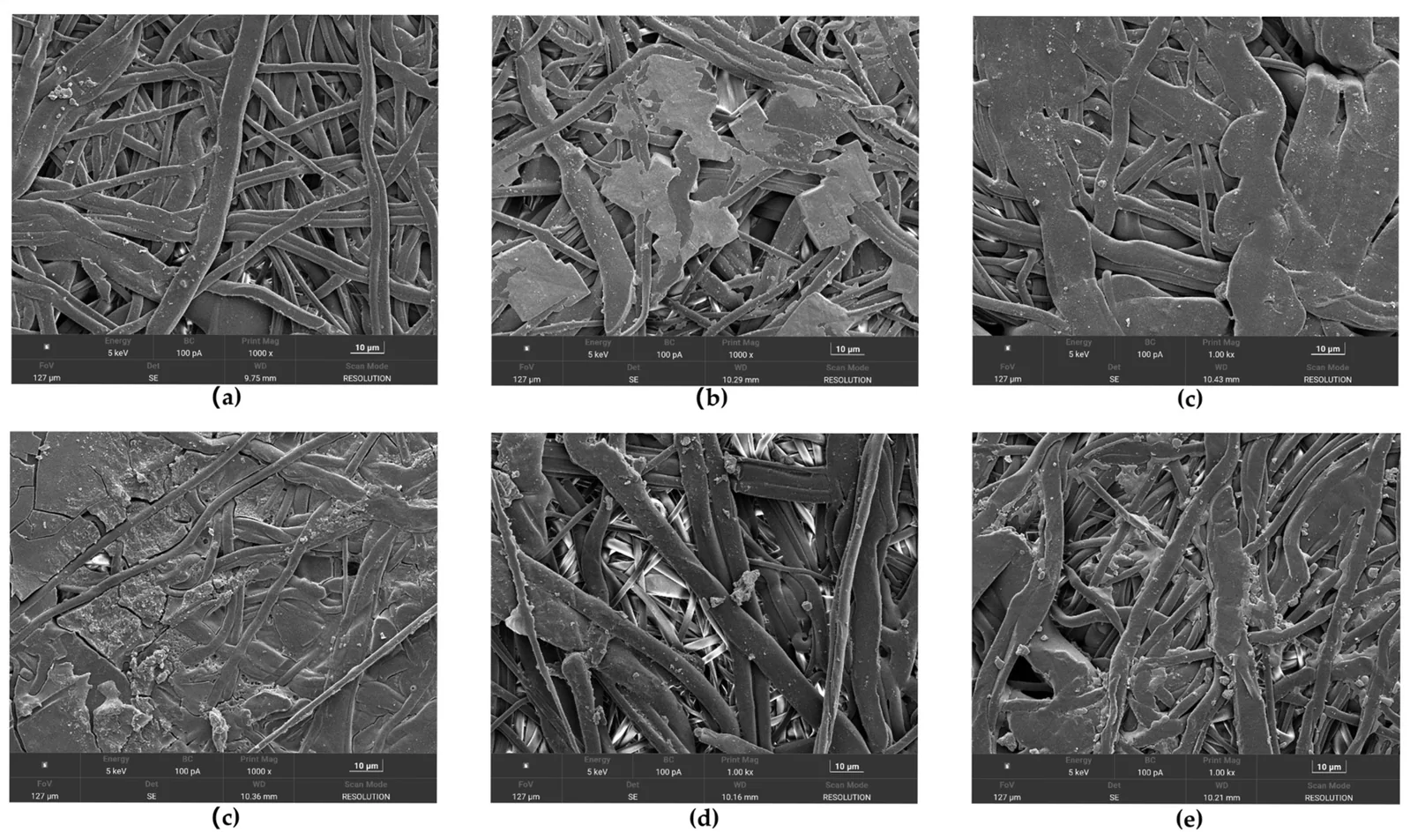

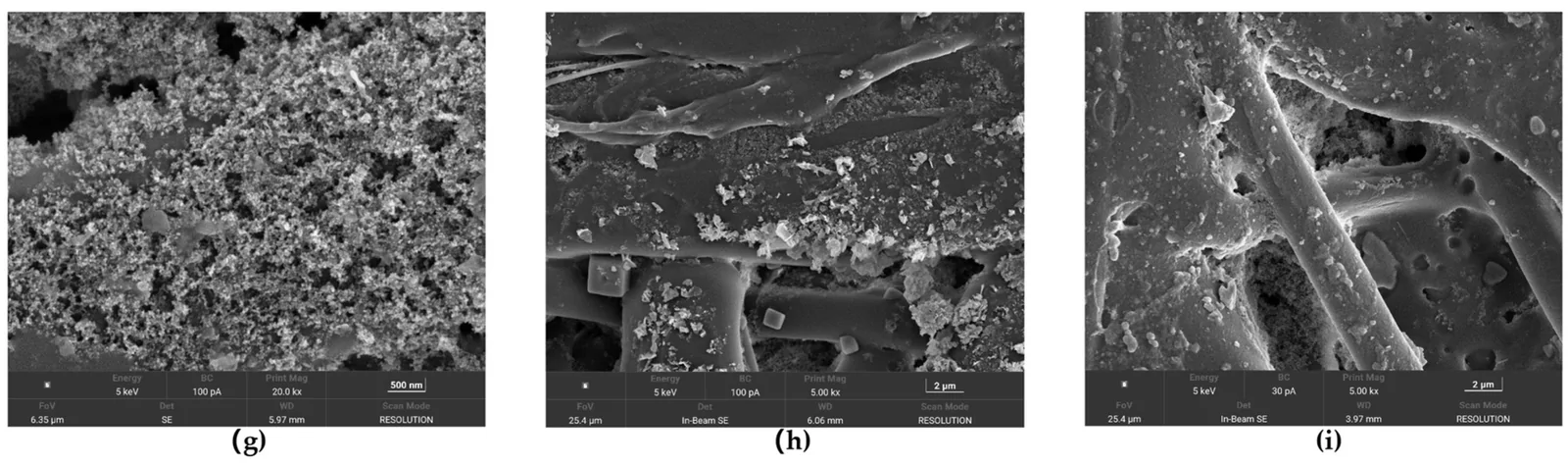
SEM surface morphology of the three membranes before and after operation. (**a**) Original membrane (1000×). (**b**) Original membrane after NaCl (1000×). (**c**) Original membrane after brackish water (1000×). (**d**) TA-modified membrane (1000×). (**e**) TA-modified membrane after NaCl (1000×). (**f**) TA-modified membrane after brackish water (1000×). (**g**) SBS/SiO_2_ superhydrophobic membrane (20,000×). (**h**) SBS/SiO_2_ superhydrophobic membrane after NaCl (20,000×). (**i**) SBS/SiO_2_ superhydrophobic membrane after brackish water (20,000×).

As shown in Figure 9a–c, after exposure to simulated brackish water, the surface of the unmodified commercial PP membrane was covered with a dense layer of inorganic crystals, forming continuous granular and lamellar structures. Such extensive coverage severely obscured the fibrous porous network, resulting in blocked channels.

(2) TA-modified membrane: discrete aggregates and hydration repulsion

In sharp contrast to the unmodified membrane, Figure 9d–f shows that the TA-modified membrane after brackish water operation did not form a continuous dense scale layer; instead, inorganic deposits exhibited typical discrete distribution and localized agglomeration. This morphology stems from the abundant phenolic hydroxyl groups in the TA coating, which strongly chelate scaling-prone ions such as Ca^2+^ and Mg^2+^, thereby disrupting composite inorganic salt scaling on the membrane surface. More importantly, these structurally altered scale aggregates adhered only weakly to the substrate and were easily detached by the high-frequency alternating fluid shear generated by the heartbeat-mimicking pulsatile flow (A2 waveform). This morphological evidence of chemical disruption combined with physical removal provides a compelling microstructural explanation for the +1.67 positive synergistic effect reported earlier.

(3) SBS/SiO_2_ superhydrophobic membrane: micro-/nano-layer and mass transfer hindrance

As shown in Figure 9g–i, at 20,000× magnification, the SBS/SiO_2_ superhydrophobic membrane displayed a pronounced micro-/nano-rough structure. After operation in NaCl and brackish water, only scattered particulate deposits appeared, with no evidence of large-scale wetting or deep scaling. This confirms that the rough layer constructed by SiO_2_ nanoparticles successfully isolated the surface, greatly reducing the actual solid–liquid contact area. However, the SEM images also reveal that this coating is relatively thick and dense; such excessive micro-/nano-structure inevitably elongates the transmembrane diffusion path for vapor molecules, resulting in the lowest absolute flux among the three membranes across all feed systems.

(4) Correlation between SEM morphology and membrane distillation performance

SEM images of the three membranes after operation showed distinct fouling states. The commercial PP membrane featured open surface pores but lacked an anti-fouling functional layer, making it susceptible to salt deposition and pore clogging. The TA-modified membrane retained the fibrous porous structure while its surface hydrophilic functional groups altered the deposition pattern of foulants, which tended to exist as scattered local clusters. The SBS/SiO_2_ superhydrophobic membrane exhibited a rough coating structure that reduced liquid intrusion into the pores, but the thicker coating also introduced additional mass transfer resistance. Although exact pore size distributions could not be quantitatively determined via capillary flow porometry in this study, the comparative SEM analysis provides clear evidence of structural variations. Visually, the ultra-thin TA coating did not severely block the inherent micro-pores of the PP substrate, which aligns with its well-maintained high flux. In contrast, the thicker SBS/SiO_2_ layer partially obscured the surface pore openings, directly contributing to the macroscopic mass transfer resistance observed during the MD process. Future studies will incorporate quantitative porometry to further refine these morphological findings.

In terms of solution type, fouling in the NaCl system mainly consisted of granular or blocky salt precipitates with a relatively simple deposition pattern, in simulated brackish water, fouling was more complex, with deposits preferentially accumulating at fiber junctions, pore openings, and coating grooves, due to the involvement of multiple ionic species in inorganic scale formation. Compared with single NaCl solutions, brackish water more readily induces complex scaling, thereby reducing the effective evaporation area of the membrane and accelerating flux decline.

SEM results correlated well with the experimental data. In the NaCl system, both the unmodified PP and TA-modified membranes showed high water production, indicating their pore structures still offered low vapor mass transfer resistance; The lower water output of the SBS/SiO_2_ superhydrophobic membrane was due to its thicker coating and partial blockage of small pores. Additionally, the SBS/SiO_2_ membrane showed minimal change in permeate conductivity, indicating that its rough superhydrophobic surface enhances anti-wetting properties. In the brackish water system, the TA-modified membrane showed a smaller flux decline, indicating that the TA surface effectively mitigated multi-ion scaling. Although the SBS/SiO_2_ membrane reduced wetting risk, its surface roughness could still serve as sites for inorganic scale accumulation, leading to poorer flux maintenance than the TA membrane.

Therefore, different modification methods offer distinct advantages. The TA-modified membrane is better suited for inhibiting scale growth in complex ionic systems, achieving higher flux with a lower decline rate; the superhydrophobic membrane has superior wetting protection in single high-salinity systems, but its flux penalty must be weighed. SBS/SiO_2_ superhydrophobic membranes are more suitable for high-salinity systems requiring high permeate quality, but coating thickness and particle distribution must be controlled to reduce mass transfer resistance. Heartbeat-mimicking pulsatile flow reduces the boundary layer thickness on the membrane surface through periodic disturbances, but it cannot fully remove crystallized and firmly adhered foulants. Therefore, membrane surface modification and pulsatile flow enhancement must be combined. SEM characterization microscopically explained the performance differences among the membranes in Section 3 and provides a reference for further membrane surface modification and optimization of operating conditions.

## 4. Conclusions

This study proposes a strategy that combines heartbeat-mimicking pulsatile flow with membrane surface modification to overcome the severe polarization and scaling bottlenecks in conventional steady-flow MD. Based on multi-scenario tests, the core conclusions are as follows:

(1) Biomimetic pulsatile flow was observed to be a major contributor to heat and mass transfer. Analysis of the sum of squares (SS) from the orthogonal experiment indicated that the pulsation waveform accounted for 50.1% of the total variance, although formal statistical significance was limited by the small sample size. Among them, the A2 exercise waveform—characterized by high frequency and short period—performed best by periodically disrupting the thermal and concentration boundary layers. Under baseline conditions, it achieved a 17.5% average flux increase over steady flow and suppressed the permeate conductivity increase to just 25%.
(2) Membrane performance strongly depended on feed characteristics. In the desalination-dominant high-salinity NaCl system, the unmodified and ultra-thin TA membranes maintained the highest flux due to extremely low mass transfer resistance, whereas the SBS/SiO_2_ superhydrophobic membrane traded a nearly 43% flux loss for optimal anti-wetting permeate purity. However, in the scaling-dominated brackish water system, the TA hydrophilic membrane achieved the highest flux, with a decline rate (27.3%) only half that of the unmodified membrane.
(3) This reveals a positive synergistic anti-scaling mechanism between pulsatile flow and the hydrophilic coating. Quantitative cross-over analysis showed a notable positive synergistic effect of +1.67 g/(m^2^·min) between heartbeat-mimicking pulsatile flow and the TA hydrophilic membrane. The microscopic morphology analysis confirmed that the chemical chelation of the TA coating transformed the inorganic scale from a continuous, dense layer into discrete, loosely adhered aggregates, which were easily detached by the periodic high-frequency fluid shear generated by the A2 pulsatile flow. This deep synergy between “microscopic physical shear” and “macroscopic chemical repulsion” offers a promising new fluid–material collaborative strategy for overcoming the fouling bottleneck in membrane distillation for complex water treatment.

Future perspectives: Since the current study focuses on the initial boundary layer dynamics and induction stage of scaling, future studies will aim to evaluate the long-term durability (>100 h) of these coatings under continuous pulsatile flow. Additionally, rigorous statistical replicate experiments will be incorporated to further validate the synergistic anti-scaling mechanisms (e.g., statistically verifying the +1.67 synergistic flux enhancement) and assess their potential for zero-liquid discharge (ZLD) industrial applications.

## Figures and Tables

**Table 1:** Main reagents.

Reagent Name	Source	Grade	Purpose
NaCl	Tianjin Damao Chemical Reagent Partnership, Tianjin, China	AR	Feed solution preparation
Anhydrous ethanol	Tianjin Fuyu Fine Chemical Co., Ltd., Tianjin, China	AR	Membrane pretreatment, dispersing impurities
Tannic acid(TA)	Shanghai Macklin Biochemical Co., Ltd., Shanghai, China	AR	Hydrophilic modified coating
Nano-SiO_2_	Ningbo Yumu New Materials Co., Ltd., Ningbo, China	15–30 nm	Superhydrophobic coating roughness construction
SBS	Sinopec Hunan Petrochemical Co., Ltd., Yueyang, China	Industrial grade	Superhydrophobic coating binder
Ethyl acetate	Guangdong Zhongli Chemical Co., Ltd., Guangzhou, China	AR	SBS solvent
CaCl_2_·2H_2_O	Tianjin Kermel Chemical Reagent Co., Ltd., Tianjin, China	AR	Brackish water formulation
MgSO_4_	Tianjin Kermel Chemical Reagent Co., Ltd., Tianjin, China	AR	Brackish water formulation
NaHCO_3_	Tianjin Kermel Chemical Reagent Co., Ltd., Tianjin, China	AR	Brackish water formulation
Distilled water	Beijing Watsons Distilled Water Co., Ltd., Tianjin, China	—	Solvent, cold-side medium

**Table 2:** Simulated brackish water formulation (1 L).

Component	Source	Mass (g)	Ions Provided
NaCl	Tianjin Damao Chemical Reagent Partnership, Tianjin, China	6.00	Na^+^, Cl^−^
CaCl_2_·H_2_O	Tianjin Kermel Chemical Reagent Co., Ltd., Tianjin, China	0.80	Ca^2+^
MgSO_4_	Tianjin Kermel Chemical Reagent Co., Ltd., Tianjin, China	0.60	Mg^2+^, SO_4_^2−^
NaHCO_3_	Tianjin Kermel Chemical Reagent Co., Ltd., Tianjin, China	0.30	HCO_3_^−^
Distilled water	Beijing Watsons Distilled Water Co., Ltd., Beijing, China	Dilute to 1 L	——

**Table 3:** Factors and levels of the orthogonal experiment.

Level	A Pulsation Parameter	B Temperature (°C)	C NaCl Concentration (g/L)
1	Resting waveform (0.8 s period/0.1 s pause)	60	10
2	Exercise waveform (0.4 s period/0.1 s pause)	70	35
3	Arrhythmia waveform (0.8 s period/0.3 s pause)	80	60

**Table 4:** Parameters of three heartbeat-mimicking pulsatile waveforms.

Waveform Name	Total Cycle Duration (s)	Cycle Pause (s)	Complete Cycle (s)	Number of Segments	Frequency Characteristics
A1 Resting waveform	0.8	0.1	0.9	6	Approx. 1.1 Hz
A2 Exercise waveform	0.4	0.1	0.5	5	Approx. 2.0 Hz
A3 Arrhythmia waveform	0.8	0.3	1.1	6	Approx. 0.9 Hz

**Table 5:** Performance indicators.

Indicator	Formulas	Notes
Membrane flux J (g/m^2^·min)	$J=\frac{\Delta m}{A\;\times \;\Delta t}$	Δm is the permeate mass increment (g), A = 0.0154 m^2^, Δt is the time interval (min). It should be noted that due to the 0.5 g resolution of the electronic balance and the fixed 15-min interval, the calculated transient flux values are inherently quantized.
Average flux (g/m^2^·min)	$\bar{J\;}=\frac{M}{A\;\times \;T}$	M is the total permeate mass, T is the total time.$\bar{J}$ reflects the flux level over the entire operating cycle.
Flux decline rate (%)	$(J_{0}\;-{\;J}_{t})/J_{0}\;\times \;100\%$	$J_{0}$ is the initial flux, and $J_{t}$ is the flux at a given time.
Water production ratio (GOR)	$\mathrm{GOR}=\frac{m_{p}\cdot \Delta H_{v}}{m_{f}\cdot C_{p}\cdot {(T}_{f,\mathrm{in}}-{\;T}_{f,\mathrm{out}})}$	$m_{p}$ is the permeate mass flow rate (kg/s), $\Delta H_{v}$ is the latent heat of vaporization of water (kJ/kg), $m_{f}$ is the feed mass flow rate (kg/s), $C_{p}$ is the feed specific heat capacity (kJ/(kg·°C)), and $T_{f,\mathrm{in}}$ and $T_{f,\mathrm{out}}$ are the hot-side inlet and outlet temperatures (°C). GOR measures the fraction of input heat used for evaporation; a higher value indicates better thermal efficiency.

**Table 6:** Performance comparison of steady and pulsatile conditions.

Condition	Average Flux (g/m^2^·min)	Initial Flux (g/m^2^·min)	Final Flux (g/m^2^·min)	Flux Decline Rate	Initial Conductivity (μS/cm)	Final Conductivity (μS/cm)	Conductivity Increase (%)
Steady 150 rpm	16.94	9.74	16.23	-	6.68	12.88	92.8%
A2 exercise pulsation	19.91	35.71	22.73	36.3%	6.68	8.36	25.1%

**Table 7:** Orthogonal design and average flux.

No.	A Pulsation Parameter	B Temperature (°C)	C NaCl Concentration (g/L)	Average Flux (g/m^2^·min)
1	A1 Resting waveform	B1 60	C1 10	30.30
2	A1 Resting waveform	B2 70	C3 60	32.47
3	A1 Resting waveform	B3 80	C2 35	26.51
4	A2 Exercise waveform	B1 60	C3 60	30.30
5	A2 Exercise waveform	B2 70	C2 35	33.01
6	A2 Exercise waveform	B3 80	C1 10	38.42
7	A3 Arrhythmia waveform	B1 60	C2 35	22.73
8	A3 Arrhythmia waveform	B2 70	C1 10	24.35
9	A3 Arrhythmia waveform	B3 80	C3 60	33.55

**Table 8:** ANOVA of the orthogonal experiment.

Source	Sum of Squares (SS)	Degrees of Freedom (df)	Mean Square (MS)	F Value	Significance
A Pulsation parameter	75.00	2	37.50	0.97	--
B Temperature	38.51	2	19.26	--	--
C NaCl Concentration	36.18	2	18.09	0.47	--
Error	38.51	2	19.26		
Total	149.69	8			

**Table 9:** Summary of orthogonal experiment data.

No.	Experimental Conditions	Initial Flux (g/m^2^·min)	Final Flux (g/m^2^·min)	Decline Rate (%)
1	A1 B1 C1	38.96	21.65	44.4
2	A1 B2 C3	45.45	21.65	52.4
3	A1 B3 C2	34.63	17.31	50.0
4	A2 B1 C3	38.96	21.65	44.4
5	A2 B2 C2	43.01	32.93	23.4
6	A2 B3 C1	43.29	34.63	20.0
7	A3 B1 C2	19.48	22.43	−15.1
8	A3 B2 C1	45.45	15.15	66.7
9	A3 B3 C3	38.96	21.65	44.4

**Table 10:** Performance comparison of the three membranes under optimal conditions.

Performance Indicator	Unmodified PP Membrane	TA Hydrophilic Modified Membrane	SBS/SiO_2_ Superhydrophobic Modified Membrane
Total permeate mass (g)	41.00	40.00	23.50
Average flux (g/m^2^·min)	44.37	43.29	25.43
Initial flux (g/m^2^·min)	54.11	49.78	30.30
Final flux (g/m^2^·min)	34.63	34.63	25.97
Flux decline rate	36.0%	30.4%	14.3%
Initial conductivity (μS/cm)	10.39	10.12	10.28
Final conductivity (μS/cm)	13.74	13.60	10.91
Conductivity increase	32.2%	34.4%	6.1%

**Table 11:** Comparison of heat transfer efficiency in the NaCl system (60 g/L NaCl, 80 °C, A2 pulsation).

Membrane Type	Hot Inlet (°C)	Hot Outlet (°C)	Sensible Heat Loss (kW)	Effective Evaporation Heat (kW)	GOR	STEC (kWh/m^3^)
Unmodified PP	79.3	67.4	0.211	0.026	0.124	5242
TA-modified	79.4	66.4	0.231	0.026	0.111	5856
SBS/SiO_2_ superhydrophobic	79.7	66.2	0.240	0.015	0.063	10,317

Note: Temperatures are the average values from the last 15 min (45–60 min) of each run.

**Table 12:** Performance comparison of the three membranes under brackish water conditions.

Performance Indicator	Unmodified PP Membrane	TA Hydrophilic Modified Membrane	SBS/SiO_2_ Superhydrophobic Modified Membrane
Total permeate mass (g)	50.50	51.50	44.00
Average flux (g/m^2^·min)	36.44	37.16	31.75
Initial flux (g/m^2^·min)	51.95	47.62	51.95
Final flux (g/m^2^·min)	23.83	34.63	25.97
Flux decline rate	54.1%	27.3%	50.0%
Initial conductivity (μS/cm)	11.88	18.10	18.05
Final conductivity (μS/cm)	16.00	20.02	20.18
Conductivity increase	34.7%	10.6%	11.8%

**Table 13:** Comparison of heat transfer efficiency in the simulated brackish water system (TDS ≈ 7.7 g/L, 80 °C, A2 pulsation).

Membrane Type	Hot Inlet (°C)	Hot Outlet (°C)	Sensible Heat Loss (kW)	Effective Evaporation Heat (kW)	GOR	STEC (kWh/m^3^)
Unmodified PP	78.4	67.7	0.191	0.020	0.106	6132
TA-modified	78.3	67.9	0.185	0.022	0.119	5462
SBS/SiO_2_ superhydrophobic	77.9	67.7	0.180	0.015	0.104	6250

Note: Temperatures are the average values from the last 15 min (75–90 min) of each run.

**Table 14:** Summary of key performance indicators for the four experimental groups.

Performance Indicator	Unmodified + Steady	Unmodified + Pulsatile	TA-Modified + Steady	TA-Modified + Pulsatile
Total permeate mass (g)	29.50	41.0	27.0	40.00
Average flux (g/m^2^·min)	31.97	44.37	29.22	43.29
Initial flux (g/m^2^·min)	34.63	54.11	34.63	49.78
Final flux (g/m^2^·min)	30.30	34.63	25.97	34.63
Flux decline rate	12.5%	36.0%	25.0%	30.4%
Initial conductivity (μS/cm)	7.77	10.39	7.65	10.12
Final conductivity (μS/cm)	9.16	13.74	9.05	13.60
Conductivity increase	17.9%	32.2%	18.3%	34.4%

**Table 15:** Synergistic effect calculation.

Effect Type	Calculation (g/m^2^·min)	Result (g/m^2^·min)
Individual effect of pulsation (Δ_1_)	J_1_ − J_0_ = 44.37 − 31.97	+12.40
Individual effect of modification (Δ_2_)	J_2_ − J_0_ = 29.22 − 31.97	−2.75
Combined effect (Δ_3_)	J_3_ − J_0_ = 43.29 − 31.97	+11.32
Synergistic effect	Δ_3_ − (Δ_1_ + Δ_2_)	+1.67

**Table 16:** Comparison of heat transfer efficiency among the four experimental groups.

Condition	Hot Inlet (°C)	Hot Outlet (°C)	Sensible Heat Loss (kW)	Effective Evaporation Heat (kW)	GOR	STEC (kWh/m^3^)
Unmodified + Steady	78.6	73.2	0.096	0.019	0.198	3283
Unmodified + Pulsatile	79.3	67.4	0.211	0.026	0.124	5242
TA-modified + Steady	78.8	73.5	0.094	0.017	0.185	3514
TA-modified + Pulsatile	79.4	66.4	0.231	0.026	0.111	5856

## Data Availability

The raw data supporting the conclusions of this article will be made available by the authors on request.
